# National Identity and Voting in a Stateless Region: Evidence From Kurdistan

**DOI:** 10.1177/01461672251370785

**Published:** 2025-09-28

**Authors:** Lewend Mayiwar, Narin Khalida Akrawi

**Affiliations:** 1 BI Norwegian Business School, Oslo, Norway; 2 Salahaddin University, Erbil, Kurdistan Region of Iraq

**Keywords:** social identity, voter participation, trust in government, life satisfaction, political behavior, Kurdish

## Abstract

Voting is a pillar of democracy, yet its psychological correlates remain understudied outside the West. We examine how national identity predicts voting among Kurds in the Kurdistan Region of Iraq, a politically autonomous yet stateless region with a long history of ethnic persecution and struggles for recognition. Voter turnout has remained high in regional Kurdish elections but has dropped to a historical low in Iraqi parliamentary elections, reflecting public disillusionment. Using a large, representative sample (*N* = 1,072), we found that national identity predicted both intended voting in the then-upcoming Kurdish elections and actual participation in the 2021 Iraqi elections. These associations also occurred indirectly via trust in government and life satisfaction and held after controlling for various psychological and sociodemographic variables. These findings suggest that a strong national identity may help sustain civic engagement even amid ongoing political and economic instability, and uncertainty over the region’s future.

## Introduction

Despite the global spread of democracy, voter turnout has steadily dropped in many countries. This trend risks undermining the extent to which elections reflect public interest and weakening government responsiveness to citizens’ needs. Traditional explanations focusing on factors such as income do not fully account for these declines (Kostelka & Blais, 2021). Problems faced by the collective, like declining voter turnout, require an understanding of the social–psychological processes that drive civic engagement, particularly those tied to group identity and institutional trust.

In recent years, researchers and policymakers have increasingly adopted a social identity perspective to address a range of societal issues (Cruwys et al., 2025). In particular, national identity has been found to play a powerful role in prosocial, collective behaviors and civic engagement (Cichocka & Cislak, 2020; Cislak et al., 2021; Golec de Zavala, 2024; Leach et al., 2023; Marchlewska et al., 2022; Rupar et al., 2021; Van Bavel et al., 2022). However, most of these studies have been conducted in Western, Educated, Industrialized, Rich, and Democratic (WEIRD; Henrich et al., 2010) contexts. Thus, research is needed to test the link between national identity and voting in non-WEIRD societies shaped by historical repression and marginalization, where ongoing political and economic instability and falling political trust in such regions (Sika, 2020) risk further eroding civic engagement. In addition, few studies have examined potential psychological mechanisms (Abou-Ismail et al., 2023; Acar & Uluğ, 2022).

In the current study, we examine whether national identity predicts voting in Kurdistan Region of Iraq (KRI), a stateless region with a history of ethnic cleansing, ongoing struggles for autonomy and recognition, and persistent regional economic and political instability. To better understand the relationship between national identity and voting in this context, we also explore trust in government and life satisfaction as indirect pathways. In KRI, voter turnout in Iraqi parliamentary elections has declined, which is concerning given the region’s reliance on the Iraqi federal government for resources and recognition. An important question, then, is whether national identity in this context can motivate voting despite persistent political uncertainty, economic hardship, and disillusionment with governing institutions.

Taken together, we contribute to both the generalizability and theoretical understanding of the link between national identity and civic engagement. First, we test this association among Kurds residing in KRI, where the formation and motivational role of national identity may differ from that of established nation-states, and where voting might be less about individual political choice and more about expressing one’s national identity. Second, we examine trust in government and life satisfaction as potential pathways that link national identity to voting. More broadly, we﻿ also contribute to a better understanding of the social psychology of the Kurdish people in KRI, which remains very limited, with a few exceptions (e.g., Buunk & Dijkstra, 2015).

### (Secure) National Identity and Civic Engagement

Social identity fulfills our basic human needs for belonging and acceptance (Tajfel & Turner, 1986). One particular form of social identity that has implications for collective prosocial behavior and civic engagement is national identity. Researchers have distinguished between two main types of national identity: an unhealthy type that is defensive and non-secure, and a healthy type of national identity that is secure and stable.

The former, often operationalized as collective narcissism, is characterized by an inflated sense of national pride and the belief that one’s group is exceptional and deserves special treatment (Cislak & Cichocka, 2023). Individuals with a defensive national identity may be more likely to endorse conspiracy theories and engage in anti-democratic political behavior (Sternisko et al., 2020). Defensive national identity has even been associated with instrumentalizing one’s group for personal gain (Cichocka et al., 2022), and studies suggest that collective narcissists may even conspire against their own in-group members to enhance national superiority (Biddlestone et al., 2022).

In contrast, a secure national identity reflects a healthy and non-defensive attachment to one’s nation, grounded in openness, trust, and concern for the collective good (Cichocka & Cislak, 2020; Cislak et al., 2021; Hamer et al., 2018). It has been consistently linked to prosocial collective behavior across various domains, such as support for government public health policies and climate change policies (Cislak et al., 2021; Golec de Zavala, 2024; Marchlewska et al., 2022; Van Bavel et al., 2022). For instance, a global collaborative project spanning 67 countries found that national identity was the strongest predictor of support for government policies on public health behaviors during COVID-19 (Van Bavel et al., 2022). Cislak et al. (2021) found that secure national identity predicted support for policies designed to mitigate climate change, while Gulevich and Osin (2025) found that national identification was positively associated with support for government-approved military actions, in contrast to national narcissism, which was linked to broader support for military aggression.

Of particular relevance to the present study, research has shown that national identity predicts voting behavior (e.g. Huddy & Khatib, 2007). Indeed, participation in elections, or voting, can be understood as an expression of group belonging and collective responsibility (Huddy & Khatib, 2007). Moreover, national identity has been conceptualized as an affective dimension of citizenship (May, 2023); in contexts like Kurdistan where identity and citizenship are deeply rooted in historical and emotional narratives, the link between identity and voting may be especially strong. However, this relationship likely depends on the form national identity takes. Rupar et al. (2021) distinguished between constructive patriotism, conventional patriotism, and glorification. Across three studies consisting of Polish samples, they found that only constructive patriotism, that is, a secure form of national identity, was positively associated with voting behavior. Rupar et al. (2024) replicated this pattern across different European contexts and different contexts, including state preliminary elections in Poland and Spain, presidential elections in Croatia, and elections for the EU parliament in Poland, the Czech Republic, and Croatia.

Given secure national identity’s consistent positive association with institutional support and civic engagement, we focus on this specific form of identity, predicting that it is positively associated with voting, even in a context where economic and political uncertainties loom large.

### Trust in Government and Life Satisfaction as Pathways

Trust in government and life satisfaction may be potential pathways that link national identity to voting. First, there is suggestive evidence that secure national identity is associated with higher government trust. As mentioned in the preceding section, strong national identification has been found to predict support for various government policies and actions (e.g. Cislak et al., 2021; Gulevich & Osin, 2025; Van Bavel et al., 2022). It is also linked to greater support for democratic principles (Marchlewska et al., 2022). Paolini et al. (2022) provided direct evidence for an association between national identity and institutional trust in a sample from Italy during the COVID-19 lockdown. Trust in government, in turn, may predict greater political engagement like voting as it reinforces perceptions that the political system is fair, responsive, and legitimate (Hetherington, 1998).

National identity might also be positively related to life satisfaction. Although empirical work is limited, there is reason to expect such an association. Social identities satisfy fundamental psychological needs that underlie life satisfaction and overall well-being (Greenaway, Cruwys et al., 2015). Empirical evidence shows that a secure national identity is associated with better mental health, partly through enhanced perceived control and reduced cynicism (Greenaway, Haslam et al., 2005; Vignoles et al., 2021). In contrast, defensive national identity has been linked to frustrated basic psychological needs and compensatory strategies (Cislak & Cichocka, 2023; Eker & Cichocka, 2024; Marchlewska et al., 2024). Thus, national identity might be directly related to life satisfaction but also indirectly via trust in government. That is, when individuals feel securely attached to their nation, they may be more inclined to see governmental institutions as reliable, which can provide a sense of certainty and control, both of which are central to psychological well-being (Helliwell et al., 2014).

Supporting this idea, research from various contexts has documented a positive link between trust in government and higher life satisfaction and lower psychological distress (e.g. Lindstrom & Mohseni, 2009). Paolini et al. (2022) conducted a study in Italy during the COVID-19 pandemic and found that national identity was associated with greater trust in political actors, which in turn was positively associated with well-being, although, somewhat paradoxically, it was also positively associated with distress. Although most of this work is based on Western samples, one exception is a study by Acar and Uluğ (2022) conducted in Turkey, an authoritarian context. They found that higher political trust was positively associated with well-being among opposition voters.

Finally, psychological well-being, particularly life satisfaction, has been widely linked to voting (e.g. Flavin & Keane, 2012; Liberini et al., 2017; Weitz-Shapiro & Winters, 2011). Life satisfaction reflects a stable, overall sense of well-being that shapes how individuals engage with their broader social environment (Diener et al., 1985). People who are more satisfied with their lives may be more inclined to invest in their communities, including through civic actions like voting (Ojeda, 2015). Although some research suggests a bidirectional relationship between life satisfaction and voting, several quasi-causal studies indicate that life satisfaction tends to precede voting (Liberini et al., 2017; Weitz-Shapiro & Winters, 2011).

In summary, we test whether a secure and healthy form of national identity is positively associated with voting via trust in government and life satisfaction. While some of these relationships have been examined in isolation in previous work, such as the association between national identity and civic engagement (Rupar et al., 2021) or the role of institutional trust in well-being (e.g. Helliwell & Huang, 2008), these have not been tested together in a unified model.

#### Potential Moderating Role of Political Efficacy

Research points to political self-efficacy (individuals’ confidence in their own political competence) as a potentially important moderator of our proposed indirect association between national identity and voting. Internal political efficacy, in particular, has been identified as one of the most important predictors of political engagement (Caprara et al., 2009; Niemi et al., 1991; Romanova & Hutchens, 2024).

Of particular relevance is a study by Acar and Uluğ (2022) which examined whether political efficacy moderates the indirect association between political trust and well-being via collective action participation in Turkey, hypothesizing that higher efficacy would amplify this relationship. However, they found no evidence of moderated mediation, suggesting that in highly authoritarian contexts, identity-based processes may overshadow the influence of political self-efficacy. Building on Acar and Uluğ (2022)’s study, we test whether political self-efficacy moderates the indirect association between national identity and voting via trust in government and life satisfaction. We had no clear prediction regarding the moderating role of political efficacy. We assumed that political efficacy might either strengthen the indirect associations between national identity and voting, or that individuals with high efficacy are less dependent on external validation from government institutions.

### Identity and Voting in Kurdistan

#### The Kurds and the Kurdish Identity

The Kurdish people are one of the world’s largest ethnic groups without a state (Mansfield, 2014) and have endured over a century of systemic state-led ethnic oppression, including genocides, and ongoing struggles for self-determination (Bozarslan et al., 2021). A common saying among Kurds, “*no friends but the mountains*,” reflects this long and difficult history, including repeated abandonment by regional and international powers. Their national identity is thus deeply rooted in narratives of resilience and the pursuit of a better future. Such narratives help shape group agency and guide collective behavior (László, 2014; Liu & Hilton, 2005; Reicher & Hopkins, 2001). National identity, especially when formed in response to historical or ongoing external threats, may motivate engagement in civic engagement even amid political instability and economic uncertainties. Indeed, studies have found that existential threats can increase cooperation even with outgroups when collective survival is at stake (Berendt et al., 2024).

Kurdistan is divided across four states: Iraq, Turkey, Iran, and Syria. Kurdish communities have faced recurring patterns of state-led repression across these contexts. In Turkey, Kurdish language and cultural expression were banned for decades, and public use of Kurdish (e.g. in schools and broadcasting) remains heavily restricted. In Iran, Kurdish identity has been marginalized through the exclusion of Kurdish literature and history from official curricula and the failure to implement constitutional language rights (Mofidi and Aghapouri, 2023). In Iraq, Kurds were subjected to brutal campaigns of violence and forced displacement, most notably the 1988 Anfal genocide under Saddam Hussein that killed around 180,000 people (Baser & Toivanen, 2017; European Parliament, 2013). The 2003 U.S.-led invasion of Iraq and Saddam’s fall opened the door to greater Kurdish autonomy in the form of a federal region, as recognized by the Iraqi constitution, but also brought new instability and power struggles to the region. Moreover, the rise of the Islamic State of Iraq and Syria (ISIS) in the 2010s posed an existential threat to Kurdish regions, reinforcing a sense of national solidarity.

In the current study, we examine the link between national identity and voting, specifically among Kurds in the KRI, where the Kurdistan Regional Government is based.

#### KRI and the Electoral Context

KRI, home to around six million people, is a constitutionally recognized autonomous region with its own government, independent parliament, and political institutions that hold periodic elections.. However, its autonomy is constrained by economic dependencies and complex relations with the central federal Iraqi government, including other neighboring states (Acar et al., 2025; Bozarslan et al., 2021).

KRI operates a parliamentary system with a 100-seat legislature elected through proportional representation (at the time of data collection in 2022, the legislature had 111 seats, which was later reduced to 100). Major political parties in the region include the Kurdistan Democratic Party (KDP) and the Patriotic Union of Kurdistan (PUK), alongside smaller parties such as the New Generation Movement, the Kurdistan Islamic Union, and parties representing minority groups. Moreover, although KRI parliamentary elections are scheduled every 4 years, they have been delayed in recent years due to political disputes and regional instability. The Kurdish election originally slated for October 2022 was postponed to 2024, with a turnout of 72%. In contrast, the 2021 Iraqi parliamentary elections saw an overall turnout drop to a historic low of 41% (Chulov, 2021). This decline has been widely linked to growing distrust toward the Iraqi federal government and broader economic dissatisfaction, particularly among youth. Among Kurds, additional factors such as unpaid public-sector salaries in KRI have also contributed to the decline in voter turnout in the Iraqi elections (M. Baban, 2025).

Notably, KRI has a predominantly young and working-age population. According to the 2024 census, 63.7% of the Kurdistan Region’s population falls within the working-age bracket of 15–64 years (H. Baban, 2024). For this demographic, political participation may be particularly driven by concerns over economic stability and long-term prospects. Given the region’s high unemployment and economic uncertainties, younger voters may rely heavily on collective identities, such as national identity, to maintain civic engagement, especially when institutional trust is low.

Voting among Kurds is widely seen as a civic duty and an expression of Kurdish national identity, consistent with the idea that national identity is constructed and expressed through collective political action (Reicher & Hopkins, 2001). A notable example came in the 2017 independence referendum, where over 92% of voters supported secession from Iraq. While the referendum was non-binding, it served as a powerful symbolic expression of Kurdish national identity. The referendum triggered strong backlash from the Iraqi federal government and neighboring states, but for many Kurds, the act of voting itself reaffirmed collective aspirations for recognition and self-determination.

A large-scale global study across 67 countries found that KRI ranked among the top 15 regions where national identity was most strongly associated with support for public health policies. A re-analysis of the dataset (for more information about the complete dataset and access, see Azevedo et al., 2023) further revealed that while secure national identity was the strongest predictor of public health compliance in KRI, defensive national identity also predicted prosocial behaviors, suggesting that even those with a more defensive orientation engaged in civic actions that served broader collective interests.

Other studies in similarly politically less stable societies the Middle East have also found that the relationship between national identity and civic engagement can diverge from patterns observed in Western societies. For example, Abou-Ismail et al. (2023) found that in Lebanon, sectarian narcissism was associated with intergroup hostility, whereas national narcissism promoted efforts toward national unity and civic cooperation.

## The Present Study

In the present study, we examine (a) whether national identity is positively associated with voting, (b) whether such a relationship might occur indirectly via trust in government and life satisfaction. As an exploratory analysis, we examine political self-efficacy as a potential moderator. We test these relationships in a large and representative sample of Kurds residing in KRI.

Our interest in national identity as a predictor of civic behavior was informed by data we gathered in KRI as part of a global collaborative project that examined the social psychological predictors of public health compliance during COVID-19 pandemic (Van Bavel et al., 2022). Among 67 countries represented in that project, KRI ranked in the top 14 for the strength of the relationship between secure national identity (as measured in the current study) and public health behaviors as well as endorsement of policy interventions, suggesting that national identity plays an important role in shaping collective-oriented behaviors and civic engagement in KRI. In that dataset, we also found a positive and moderately strong correlation between national identity and psychological well-being.

Our outcome variables in the present study focused on voting in both the Kurdish and Iraqi parliamentary elections. This allowed us to test whether individuals with stronger national identity were more likely to vote in regional elections, as well as in central government elections where turnout has been much lower. Specifically, we measured: (a) intention to vote in the upcoming (originally scheduled for 2022) Kurdish parliamentary election, and (b) self-reported participation in the 2021 Iraqi parliamentary elections. For the Iraqi elections, we measured whether respondents had voted in the most recent election, as asking about intentions for a future election was impractical given the long interval until the next one (2025). In contrast, we assessed voting intention for the Kurdish parliamentary elections because the election was scheduled to occur within months of the survey, although it was later postponed.

## Openness and Transparency Statement

We report how we determined the sample size, all data exclusions, and all measures in this study. Data, code, and material (original language and English-translated versions) are available on the Open Science Framework (OSF) repository: https://osf.io/mvxwb/. We use two-tailed *p*-values and confidence intervals and set α = .05. The study was not preregistered. Participants provided their consent to participate.

We performed all analyses in RStudio (R Core Team, 2023) and used the packages *psych* (v2.4.6; Revelle, 2024), *dplyr* (v1.1.4; Wickham et al., 2023), *apaTables* (v2.0.8; Stanley, 2021), *readxl* (v1.4.3; Wickham & Bryan, 2023), as well as Hayes’ PROCESS macro in R version 4.3 (Hayes, 2022).

## Method

### Sample

We aimed for a large sample of at least 1,000 respondents to obtain sufficient power for mediation tests. We recruited 1,072 respondents residing in KRI, during spring 2022. Due to missing data on one or more variables in the mediation models (identity, trust in government, life satisfaction, and the outcome variable), 73 cases were excluded from the model predicting voting intention (final *N* = 999), and 131 cases were excluded from the model predicting past voting (final *N* = 941).

We conducted a Monte Carlo sensitivity analysis for the indirect associations, using an online tool developed by Schoemann et al. (2017). We used the following parameters: α = .05, standard deviation = 1.00 for all variables, number of replications = 1,000, and number of draws = 20,000. We estimated power for a very small indirect association by using *r* = .10 for the *a* and *b* paths that make up the indirect association (0.01). The results indicated that a sample of 999 participants provides 92% power to detect an indirect association of 0.01.

Respondents were recruited through a survey/research agency (Sheekar Research; https://www.sheekar4research.com/En/Services.php) located in Erbil, KRI. To ensure as representative of a sample as possible, we targeted potential respondents from different areas of KRI with different demographic backgrounds. Table 1 summarizes the sample characteristics. Average age was 30.24 (*SD* = 10.75). 60.5% of the respondents identified as male, and 39.3% identified as female.

**Table 1.** Sample Characteristics.

Characteristics	*N*	%
Age		
18–29	592	55.2
30–39	278	25.9
40–49	133	12.4
50–59	52	4.9
60+	16	1.5
Gender		
Male	649	60.5
Female	421	39.3
Marital status		
Single	540	50.4
Married	516	48.1
Divorced/Widowed	15	1.4
Highest level of education		
Illiterate/no schooling	43	4.0
Only reading, no writing	101	9.4
Primary school	167	15.6
High school graduate	262	24.4
Post high school	195	18.2
Bachelor’s degree	286	26.7
Master’s degree	12	1.1
PhD	5	0.5
Personal income		
<500,000 IQD	600	56.0
500,000–1,000,000 IQD	426	39.7
1,500,000–2,000,000 IQD	35	3.3
2,000,000–4,000,000 IQD	7	0.7
<5,000,000 IQD	3	0.3
Occupation		
Public sector employee	181	16.9
Private sector employee	71	6.6
Business owner	53	4.9
Specialist	35	3.3
Military	47	4.4
Farmer	16	1.5
Construction	92	8.6
Unemployed	296	27.6
Student	153	14.3
Retired	11	8.7
Other	116	10.8
City		
Erbil	442	41.2
Suleymani	410	38.2
Duhok	189	17.6
Halabja	30	2.8
Area of residence		
Inner city	512	47.8
Outer city	283	26.4
Outer rings	196	18.3
Rural area/village	80	7.5

*Note.* IQD = Iraqi dinar. 500,000 IQD ≈ 380 USD, 1,000,000 IQD ≈ 764 USD, 2,000,000 IQD ≈ 1,527 USD, 4,000,000 IQD ≈ 3,055 USD, 5,000,000 IQD ≈ 3,819 USD.

### Measures

All measures were translated from English to Kurdish by a team at the research agency. We back-translated to English to ensure accuracy and conceptual equivalence.

#### Voting

We measured intention to vote in the upcoming Kurdish parliamentary election, using a three-point Likert scale (1 =* Will not vote*, 2 =* Not sure*, 3 =* Will vote*), and whether they voted in the 2021 Iraqi parliamentary election that had taken place some months prior to data collection (0 =* Did not vote*, 1 =* Voted*).

We included two additional voting-related variables for descriptive and exploratory purposes. First, we asked participants about their reasons for voting or not voting in the most recent Iraqi parliamentary election. We included a range of possible reasons for voting and abstention, reflecting issues relevant in KRI at the time. To simplify interpretation, we grouped responses into a few broader themes in our analysis, allowing us to examine whether those who voted were more likely to mention identity-related motivations (e.g. “Protecting Kurdish authority in KRI”) while those who did not vote were more likely to report distrust-related concerns (e.g. “I do not trust the government”). The results are shown in Figure 1 in the Results section (the full list of response options is available in the Supplemental Materials on OSF).

**Figure 1.**
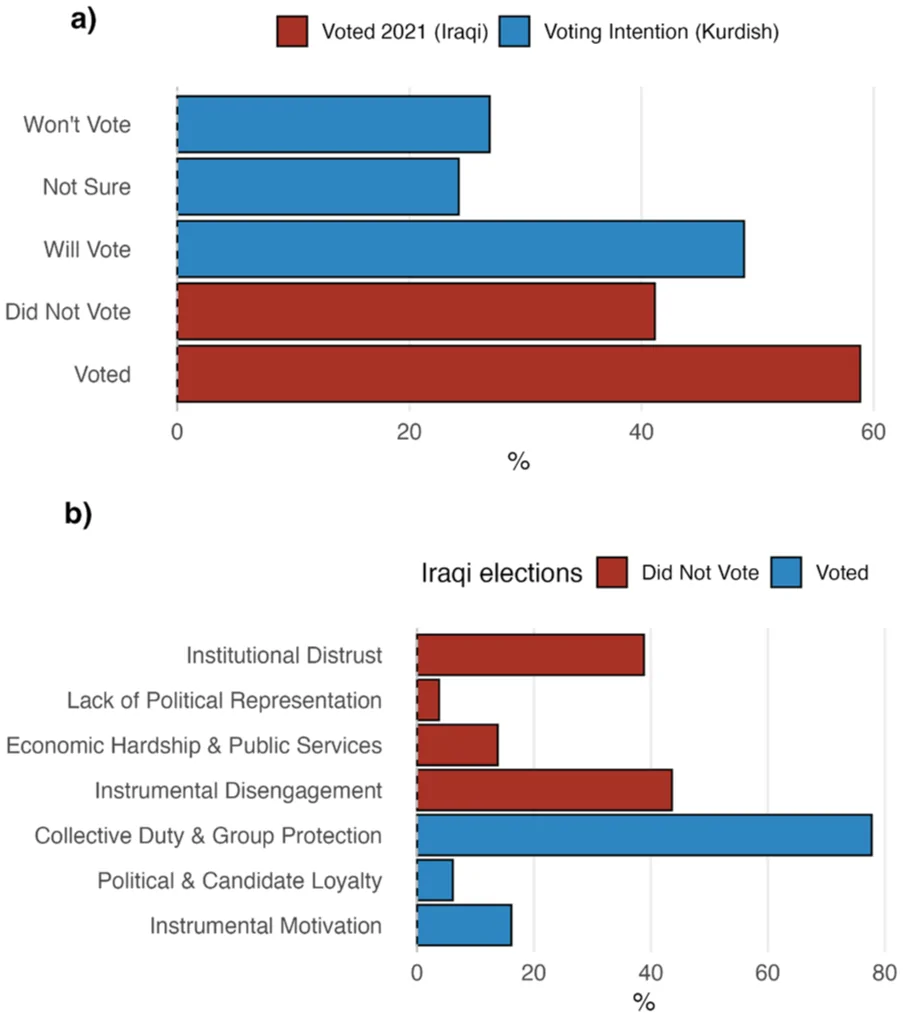
Self-reported voting in the then-upcoming Kurdish election and voting in the previous Iraqi election and self-reported reasons for actual voting/not voting in the 2021 Iraqi parliamentary election. *Note.* Panel A shows the percentage of participants who reported voting or not voting in the most recent Iraqi parliamentary elections (red bars), and their stated intentions to vote in the then-upcoming Kurdish parliamentary elections (blue bars). Panel B displays participants’ self-reported reasons for actual voting (blue bars) or not voting (red bars) in the 2021 Iraqi elections (respondents could select multiple reasons).

Second, a supplementary analysis, given previous postponements in the KRI parliamentary elections, we measured participants’ optimism regarding whether the Kurdish election would take place as planned. While optimism is typically measured as a general trait (Scheier & Carver, 1985), we measured optimism as a state, similar to previous work (e.g. Kluemper et al., 2009; Millstein et al., 2019), but here in relation to a specific political event. Optimism was measured with a single item (“Do you think the KRI parliamentary election will take place this year?”) on a three-point scale (1 =* Will be postponed*, 2 =* Not sure*, 3 =* Will happen as planned*). Results are reported in the Supplemental File on OSF (see Table S1).

#### National Identity

We measured (secure) national identification using a scale adopted from (Van Bavel et al., 2022). The scale contains two items; one from Postmes et al. (2013) and one from Cameron (2004) that measures identity centrality. We used a Kurdish version of the scale that has been used in a Kurdish sample (Van Bavel et al., 2022). The two items were “I identify as Kurdish” and “Being a Kurd is an important reflection of who I am” (α = .79). Respondents indicated their level of agreement with these statements on a three-point Likert scale (1 =* Strongly disagree*, 2 =* Neither agree nor disagree*, 3 =* Strongly agree*).^1^

#### Trust in Government

We measured trust in government using a validated nine-item scale developed by Grimmelikhuijsen and Knies (2017). The scale taps into three dimensions of trust: competence, benevolence, and integrity. Each dimension is measured using three items. Example items include “I believe that governments and governmental bodies are capable,” “I believe that governments and governmental bodies act in the interest of citizens,” and “I believe that governments and governmental bodies are honest.” Respondents indicated their level of agreement with the statements on a five-point Likert scale (1 =* Strongly disagree*, 5 =* Strongly agree*). All three dimensions were highly correlated (see Table 2). We thus averaged all items across the three dimensions and computed a single index of overall trust in government (α = .95).

**Table 2.** Means, Standard Deviations, and Correlations.

Variable	*M*	*SD*	1	2	3	4	5	6	7	8	9	10	11	12	13	14	15
1. Vote	2.19	0.83															
2. Voted	0.59	0.49	.57**														
3. NaID	2.52	0.60	.20**	.19**													
4. GT	2.75	1.04	.34**	.29**	.37**												
5. GT-B	2.69	1.16	.32**	.29**	.34**	.95**											
6. GT-I	2.62	1.16	.31**	.27**	.27**	.93**	.87**										
7. GT-C	2.92	1.05	.32**	.25**	.41**	.88**	.76**	.69**									
8. LS	2.46	0.97	.14**	.14**	.05	.15**	.16**	.15**	.10**								
9. CD	2.10	0.47	.26**	.24**	.35**	.44**	.40**	.44**	.38**	.03							
10. PSF	2.67	0.82	.12**	.12**	−.13**	.03	.01	.07*	.01	.14**	.03						
11. Rel	3.56	0.84	−.08**	−.04	.13**	−.01	.00	−.05	.03	−.06	.07*	−.24**					
12. PI	2.04	1.02	.34**	.29**	.06*	.30**	.27**	.30**	.26**	.11**	.16**	.40**	−.31**				
13. Inc	1.62	0.86	.12**	.15**	.09**	.03	.05	.03	.01	.08*	.10**	−.01	−.00	.05			
14. Edu	4.30	1.49	.05	−.02	.09**	−.03	−.00	−.08**	−.00	−.03	−.03	−.11**	.10**	−.07*	.26**		
15. Age	30.24	10.75	.03	.14**	.07*	.01	.02	.02	−.02	.02	.04	−.07*	.01	−.01	.19**	−.22**	
16. Female	1.39	0.49	−.08**	−.13**	.04	−.02	−.02	−.05	.01	−.06	−.00	−.15**	.17**	−15**	−.10**	.02	.00

*Note. M* and *SD* are used to represent mean and standard deviation, respectively. Vote = intention to vote in the then-upcoming parliamentary elections in KRI (three-point scale). Voted = whether they voted in the previous Iraqi parliamentary elections (0 = No, 1 = Yes). NaID = National identity (three-point-scale); GT = Trust in government (5-point scale); GT-C = Trust in government’s competence (five-point scale); GT-B = Trust in government’s benevolence (five-point scale); GT-I = Trust in government’s integrity (five-point scale); CD = Civic duty (three-point scale); PSF = (internal) Political self-efficacy (five-point scale); LS = Life satisfaction (five-point scale); Rel = Religiousness (four-point scale); PI = Political interest (four-point scale); Inc = Income; Edu = Education.

* *p* < .05. **p < .01.

#### Life Satisfaction

The life satisfaction item was adopted from previous research (e.g. Oswald & Wu, 2010). Respondents indicated their level of satisfaction with their life using a four-point scale on the item “In general, how satisfied are you with your life?” (1 =* Very dissatisfied*, 2 =* Dissatisfied*, 3 =* Satisfied*, 4 =* Very satisfied*).

#### Political Self-Efficacy

We measured internal political self-efficacy using a scale originally developed by the American National Election Survey (ANES) and later revised by Niemi et al. (1991). The scale consists of four items, all of which are rated on a five-point Likert scale (α = .52). Example items include “How often do politics and government seem so complicated that you can’t really understand what’s going on?” and “How much can people like you affect what the government does?”

#### Control Variables

We included several control variables that have been used in previous voting studies (Blais & Galais, 2016; Pruysers et al., 2019) as part of an additional robustness check.^2^

##### Civic Duty

We included a measure of civic duty, a well-established predictor of voting (Blais & Achen, 2019). We used the four-item Civic Duty Scale developed by Blais and Galais (2016). The items are “How strongly do you feel that voting in an election is a duty,” “How guilty would you feel if you did not vote in an election?,” “I see voting as a way to show love for my country,” and “It is OK to abstain if you have no opinion in an election.” Responses on the first item were given on a four-point Likert scale from 1 =* Voting is a choice/not a duty* to 4 =* Very strongly feel it is a duty*. Responses on the last three items were given on a scale from 1 =* Strongly disagree* to 3 =* Strongly agree*).^3^ The last item (“It is OK to abstain if you have no opinion in an election”) significantly reduced the scale’s internal consistency (from Cronbach’s α = .55 to α = .71). Given that this may reflect a distinct attitude toward abstention rather than a direct sense of duty to vote, and to ensure a more reliable measure of civic duty, we exclude it from our final scale. Our results remain the same if the item is included.

##### Political Interest

Respondents indicated their level of political interest on a four-point Likert scale (1 =* Not at all*, 4 =* A lot*).

##### Religiosity

Respondents indicated how important religion is in their life on a four-point Likert scale (1 =* Not at all important* to 4 =* Very important*).

##### Demographics

Respondents reported the following demographic variables: age, gender (0 = Male, 1 = Female), education (1 =* Illiterate/no schooling*, 2 =* Only reading, no writing*, 3 =* Primary school*, 4 =* High school*, 5 =* Post high school*, 6 =* Bachelor*, 7 =* Master’s*, 8 =* PhD*), personal income (1 =* < 500,000 IQD*, 2 =* 500,000–1,000,000 IQD*, 3 =* 1,000,000 IQD*, 4 =* 1,500,000–2,000,000 IQD*, 5 =* 2,000,000–4,000,000 IQD*, 6 =* >5,000,000 IQD*).

## Results

### Descriptives

#### Means and Correlations

Table 2 shows the means, *SD*s, and correlations of all key variables. National identity and trust in government were positively correlated with voting in the next and previous election. Moreover, trust in government was positively correlated with life satisfaction and the two voting variables. Life satisfaction was also positively correlated with both voting variables. It is also noteworthy that the average level of life satisfaction in this sample is quite low, below the midpoint value. The same goes for the average trust in government variable, particularly regarding its integrity dimension. The mean value of national identity, on the other hand, was above the scale’s midpoint value.

Overall, these descriptive results provide preliminary support for our hypothesized associations and also show that both government trust and life satisfaction are quite low, an outcome we anticipated for KRI, given the region’s ongoing political and economic instability and uncertainty.

#### Reasons for Voting and Not Voting

Figure 1 shows the proportion of self-reported voting intentions for the upcoming Kurdish elections and voting in the previous Iraqi election in 2021, and reasons for voting/not voting in the previous Iraqi election. Most respondents (46%) reported that they intended to vote in the upcoming Kurdish election. Similarly, most respondents (58%) reported that they had voted in the previous Iraqi election in 2021. The most common motivation for voting in the Iraqi election was Collective Duty & Group Protection (e.g. “Protecting Kurdish authority in KRI”), while Institutional Distrust (e.g. “I do not trust the government”) and Instrumental Disengagement (e.g. “Past votes had no impact on my life or other citizens’”) were the leading reasons for non-participation. These findings provide preliminary descriptive evidence into the importance of identity-based motivation in predicting voting.

### Main Analyses

#### Association Between National Identity and Voting

We ran a linear regression model and a multiple logistic regression model to predict intention to vote in the then-upcoming Kurdish election of 2022 (which was later postponed) and actual voting during the Iraqi election in 2021. We report odds ratios for the logistic regression model. Odds ratios can be interpreted in terms of the percent change in the likelihood of the dependent variable (voting in the last election). For example, an odds ratio of 1.5 indicates that for a one-unit change in the independent variable, the likelihood of the dependent variable (voting) occurring increases by 50%. Conversely, an odds ratio of 0.50 indicates that for a one unit change in the independent variable, the likelihood of the dependent variable (voting) occurring decreases by 50%. We *z*-standardized all continuous predictors.

We first tested the relationship between national identity and the two voting variables without control variables. National identity positively predicted voting intention in the then-upcoming Kurdish election β = 0.20, *p* < .001 (two-tailed), 95% CI [0.15, 0.26] and actual voting in the Iraqi election β = 1.46, *p* < .001 (two-tailed), 95% CI [1.28, 1.66].

Next, we ran the same two models while including the control variables (voting as a civic duty, internal political self-efficacy, political interest, income, education, religiosity, age, and gender). The coefficients from these models are shown in Figure 2. National identity positively predicted voting intention in then-upcoming Kurdish election β = 0.11, *p* < .001 (two-tailed), 95% CI [0.05, 0.17] and actual voting in the Iraqi election β = 1.25, *p* = .003 (two-tailed), [1.08, 1.45]. Civic duty was the strongest predictor of voting β = 0.35, *p* < .001 (two-tailed), [0.29, 0.41] and voting in the last election β = 2.07, *p* < .001 (two-tailed), [1.76, 2.43]. This is expected given that the civic duty scale specifically asks about their sense of duty in the context of voting.

**Figure 2.**
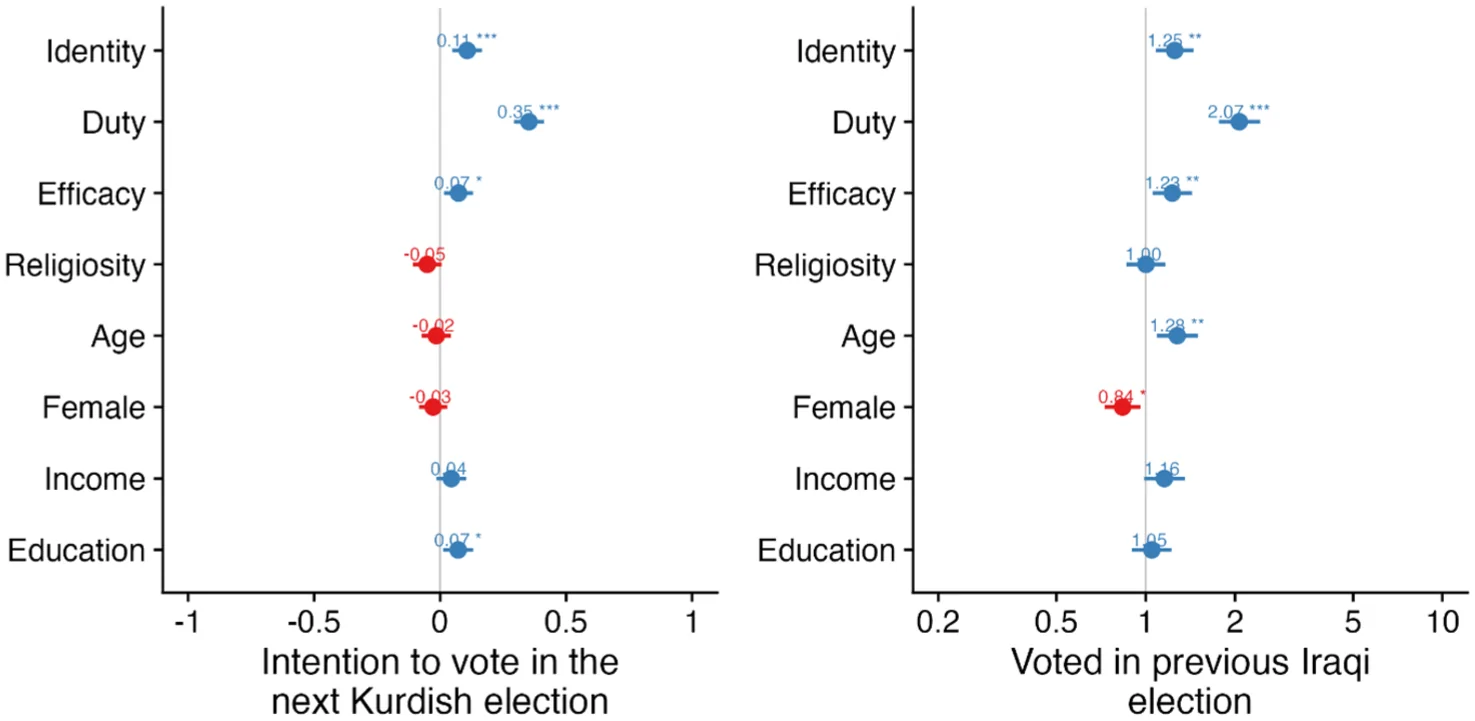
Forestplots of regression coefficients. *Note.* Coefficients are *z*-standardized. The gray vertical line at 1 is the vertical intercept denoting no association. Estimates on the right side of the gray vertical line are positively associated with voting. Estimates on the left side of the gray vertical line are negatively associated with voting. *p < .05. **p < .01. ***p < .001.

#### Indirect Associations via Trust in Government and Life Satisfaction

We tested indirect associations via trust in government and life satisfaction using the PROCESS package for R (Hayes, 2022), specifically Model 6, which allows for testing individual mediators as well as potential serial mediation. We used 5,000 boot-strapped 95% confidence intervals for the estimate of the predictors and indirect associations. An indirect association is considered significant if zero falls outside the 95% confidence interval. As a robustness check, we ran all models with the same control variables. The results remained the same, which we report in the Supplemental File on OSF (see “IdentityVotingKurdistan.html” file).

Table 3 summarizes the estimates of individual predictors on the two voting outcome variables along with the indirect association estimates. The indirect associations are shown in Figure 3. There was a significant main indirect association via trust in government but not life satisfaction. In addition, there was a significant serial indirect association via trust in government and life satisfaction. Specifically, national identity was linked to greater trust in government, which was linked to higher life satisfaction, which in turn was associated with greater voting intention. The results indicated partial mediation, as the main association between identity and voting became weaker once the mediators were included.

**Table 3.** Summary of Regression Models and Indirect Associations.

Predictor	DV: voting intention (Kurdish elections)				DV: past voting (Iraqi elections)			
	β	95% CI	*p*	*R*²	β	95% CI	*p*	*R*² (Nagelkerke)
Outcome: GovTrust				.14				.14
Constant	−.01	[−0.07, 0.04]	.644		−.01	[−0.07, 0.05]	.745	
NationID	.37	[0.31, 0.42]	<.001		.37	[0.31, 0.43]	<.001	
Outcome: LifeSat				.02				.02
Constant	.00	[−0.06, 0.06]	.933		.01	[−0.05, 0.07]	.771	
NationID	−.01	[−0.07, 0.06]	.786		.01	[−0.06, 0.07]	.868	
GovTrust	.15	[0.09, 0.22]	<.001		.14	[0.08, 0.21]	<.001	
Outcome: Vote				.13				.14
Constant	2.20	[2.15, 2.24]	<.001		.40	[0.26, 0.54]	<.001	
NationID	.09	[0.03, 0.14]	.001		.16	[0.02, 0.30]	.028	
GovTrust	.24	[0.18, 0.29]	<.001		.58	[0.42, 0.73]	<.001	
LifeSat	.08	[0.03, 0.13]	.002		.23	[0.09, 0.37]	.001	
Total Effect				0.04				.05
NationID	.17	[0.12, 0.21]	<.001		.38	[0.25, 0.50]	<.001	
Indirect associations								
Total	.09	[0.07, 0.12]	Sig.		.23	[0.17, 0.30]	Sig.	
NationID → GovTrust → Vote	.09	[0.06, 0.11]	Sig.		.22	[0.15, 0.29]	Sig.	
NationID → LifeSat → Vote	−.00	[−0.01, 0.00]	NS		.00	[−0.02, 0.02]	NS	
NationID → GovTrust → LifeSat → Vote	.00	[0.00, 0.01]	Sig.		.01	[0.00, 0.02]	Sig.	

*Note.* The indirect associations were tested using bootstrapping with 95% confidence intervals (5,000 bootstrap samples). NationID = national identity; GovTrust = trust in government; LifeSat = life satisfaction; Duty = civic duty; Sig = significant indirect association; NS = non-significant indirect association.

**Figure 3.**
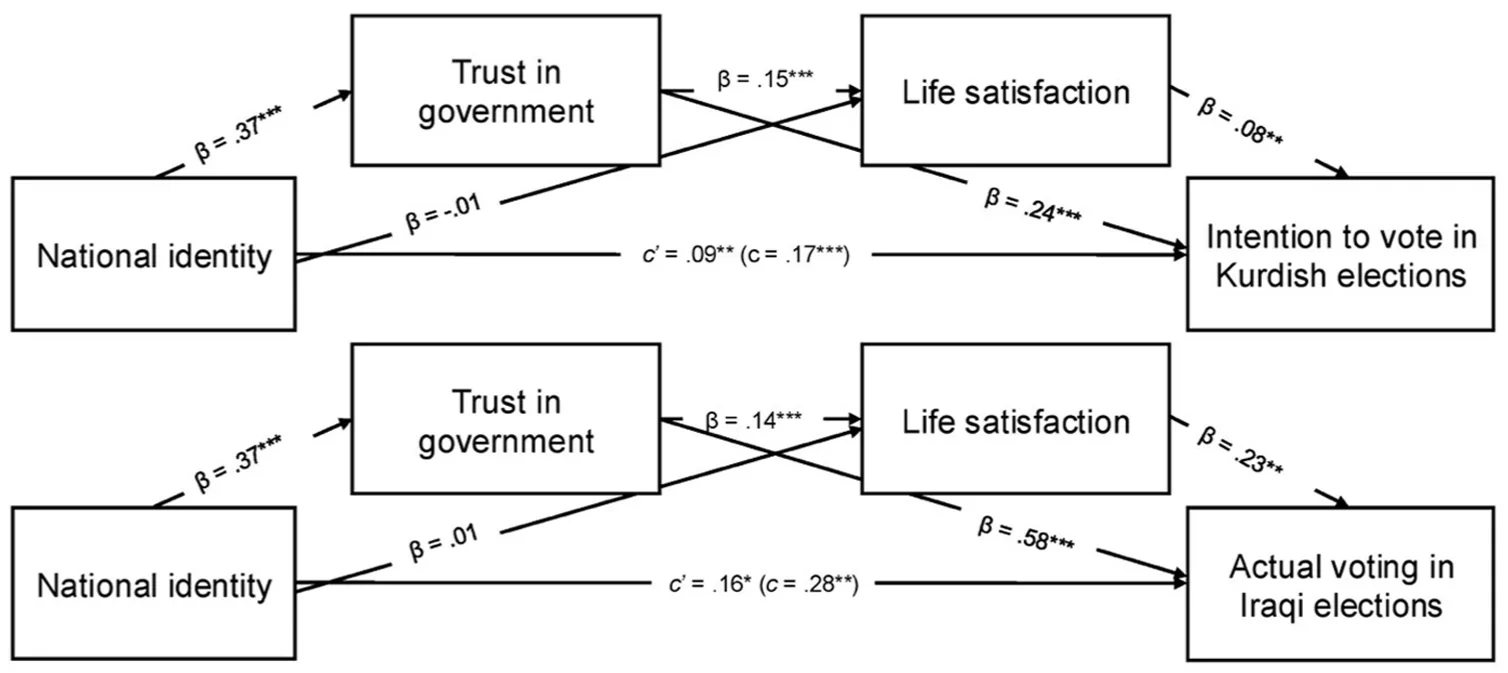
Indirect associations. *Note.* Coefficients are *z*-standardized. *c’* denotes the “direct effect” (i.e. the association between national identity and voting controlling for the mediators). *c* denotes “total effect” (i.e. the association between national identity and voting without controlling for the mediators).

Given the moderate correlation between civic duty and trust in government (*r* = .48, *p* < .001), we explored whether the indirect association of national identity on voting via trust in government would similarly emerge if we substituted civic duty (one of our control variables) for trust in government. This allowed us to test whether national identifiers’ civic engagement behaviors might be driven more by an internalized moral obligation to vote rather than trust in institutions (i.e. voting as a duty regardless of institutional trust). We found that civic duty alone mediated the relationship between national identity and both voting intention (*b* = 0.11, 95% CI [0.08, 0.14]) and past voting (*b* = 0.23, [0.17, 0.31]). However, this alternative model did not result in a serial pathway through life satisfaction (voting intention: *b* = 0.01, [−0.001, 0.004]; past voting: *b* = 0.004, [−0.002, 0.01]). This suggests that while civic duty plays an important role in linking national identity to voting, it does not account for the broader psychological pathway.

Finally, as an exploratory analysis, we examined whether the serial mediation model extended to participants’ belief that the Kurdish election would take place as planned. Interestingly, national identity was not directly associated with greater optimism but did predict optimism indirectly via the chain of trust in government and life satisfaction. This aligns with established social psychological findings that greater life satisfaction is associated with increased optimism. The results are reported in the Supplemental Material on OSF (summarized in Table S1).

#### Moderation by Political Efficacy

Finally, we explored whether internal political efficacy moderated the serial indirect association. We used “Model 83” in PROCESS, which tests for moderation of the path between the independent variable (national identity) and the first mediator (trust in government), as this was deemed most aligned with our theorizing. The results remained the same when we tested moderation of other paths. As shown in Table 4, internal political self-efficacy moderated the path between national identity and trust in government, indicating that the association was twice as strong among those with low efficacy. Moreover, the index of moderated mediation was significant, indicating that the indirect association between national identity and voting via trust in government and life satisfaction was stronger among individuals with low internal political efficacy.

**Table 4.** Summary of Moderated Serial Mediation Analysis Results.

Predictor	DV: voting intention (Kurdish elections)			DV: past voting (Iraqi elections)		
	β	95% CI	*p*	β	95% CI	*p*
Outcome: GovTrust						
Constant	−.00	[−0.05, 0.06]	.888	.01	[−0.05, 0.07]	.788
NationID	.35	[0.29, 0.40]	<.001	.35	[0.29, 0.41]	<.001
Efficacy	.13	[0.07, 0.19]	<.001	.12	[0.06, 0.18]	<.001
NationID × Efficacy	.14	[0.09, 0.19]	<.001	.14	[0.08, 0.19]	<.001
Low Efficacy (−1 SD)	.21	[0.13, 0.29]	<.001	.22	[0.13, 0.30]	<.001
High Efficacy (+1 SD)	.48	[0.42, 0.55]	<.001	.49	[0.42, 0.56]	<.001
Outcome: LifeSat						
Constant	.00	[−0.06, 0.06]	.933	.01	[−0.05, 0.07]	.771
NationID	−.01	[−0.07, 0.06]	.786	.01	[−0.06, 0.07]	.868
GovTrust	.15	[0.09, 0.22]	<.001	.14	[0.08, 0.21]	<.001
Outcome: Vote						
Constant	2.20	[2.15, 2.24]	<.001	.40	[0.26, 0.54]	<.001
NationID	.09	[0.03, 0.14]	.001	.16	[0.02, 0.30]	.028
GovTrust	.24	[0.18, 0.29]	<.001	.58	[0.42, 0.73]	<.001
LifeSat	.08	[0.03, 0.13]	.002	.23	[0.09, 0.37]	.001
Indirect path being moderated	Index of moderated mediation	Low efficacy (−1 SD)	High efficacy (+1 SD)	Index of moderated mediation	Low efficacy (−1 SD)	High efficacy (+1 SD)
NationID → GovTrust → Vote	.03 [0.02, 0.05]	0.05 [0.03, 0.07]	0.02 [0.08, 0.15]	.08 [0.05, 0.12]	0.13 [0.07, 0.19]	0.28 [0.20, 0.38]
NationID → LifeSat → Vote	NS			NS		
NationID → GovTrust → LifeSat → Vote	.002 [0.001, 0.003]	0.003 [0.001, 0.005]	0.01 [0.002, 0.01]	.004 [0.001, 0.009]	0.01 [0.002, 0.01]	0.02 [0.005, 0.03]

*Note.* The indirect associations were tested using bootstrapping with 95% confidence intervals (5,000 bootstrap samples). NationID = national identity; GovTrust = trust in government; LifeSat = life satisfaction; Duty = civic duty; NS = index of moderated mediation is not significant.

## Discussion

We tested how national identity in a stateless region is associated with voting in the KRI, where identity has been shaped by a history of repression, ethnic cleansing, and the ongoing pursuit of recognition and autonomy. Using a large, representative sample, we found that national identity positively predicted voting intention in the KRI parliamentary election and actual voting in the 2021 Iraqi parliamentary elections. We also found indirect associations via trust in government and life satisfaction for both voting measures.

It is noteworthy that our findings held both for intentions to vote in the KRI parliamentary elections, where turnout remains relatively high, and for actual voting in the Iraqi parliamentary elections, where turnout has dropped to a historical low. This suggests that national identifiers are willing to vote even in contexts where there is widespread public disillusionment that has kept voter turnout low. This shows how a shared national identity may override cynicism or discouragement stemming from broader institutional discontent.

Our findings align with the global COVID-19 findings from Van Bavel et al. (2022) that placed KRI among the top 14 regions where national identity strongly predicted public health compliance. Here, we extend these findings to a different context, namely, voting, and identify potential pathways. First, we found that national identity was positively associated with trust in government, which in turn was positively associated with voting. Second, we found a serial pathway: national identity predicted trust in government, which was positively linked to life satisfaction, which in turn was positively associated with voting. This is consistent with studies that have linked life satisfaction to voting using quasi-causal methods (Liberini et al., 2017; Weitz-Shapiro & Winters, 2011), as well as with research suggesting that individuals who are more satisfied with their lives are more likely to engage with their communities through civic acts such as voting (Ojeda, 2015). Furthermore, in line with research linking life satisfaction to hope and optimism (Bailey et al., 2007), a supplementary analysis showed that national identity predicted optimism that the then-upcoming KRI election would take place, via trust in government and life satisfaction, despite previous regional election postponements.

In contrast to trust in government, life satisfaction alone did not mediate the relationship between national identity and voting; it only did so as part of the serial pathway, with trust in government as the first link in the chain. We had expected to find an indirect association via life satisfaction as well, based on our re-analysis of the KRI dataset from the global COVID-19 project, which showed a moderately strong correlation between secure national identity and well-being. One explanation might be differences in measures: while the COVID-19 project measured broader well-being, the present study measured life satisfaction. Another possibility is that in the intensely uncertain pandemic context, national identity offered more direct emotional relief. However, these analyses were not preregistered and need to be followed up in future research.

Moreover, the indirect associations were stronger for past voting behavior than for voting intention. It is difficult to say what might have contributed to this discrepancy. On the one hand, it may reflect differences in how two outcome measures were measured and analyzed. On the other hand, it might reflect underlying psychological differences between realized and anticipated behavior: Past voting reflects a completed action tied to a known political event, whereas voting intention concerned an upcoming election that, at the time of data collection, had already been postponed and remained uncertain. In KRI, such delays are not uncommon, and repeated postponements may have contributed to a kind of “political fatigue” or cynicism, making intentions to vote more tentative. Given that behavioral intentions are shaped by perceptions of control and predictability (Ajzen, 1991), respondents may have felt unsure about whether the election would take place or whether their participation would matter, thus potentially weakening the associations with national identity, trust, and life satisfaction.

It is worth noting that this interpretation contrasts with the Social Identity Model of Collective Action (SIMCA; van Zomeren et al., 2008), which suggests that identity-based political engagement tends to increase under conditions of instability and perceived illegitimacy. From that perspective, one might expect *stronger* effects of national identity in politically uncertain environments. However, the type of uncertainty emphasized in SIMCA typically involves intergroup threat or structural disadvantage, whereas the uncertainty surrounding the Kurdish election was procedural and institutional, that is, whether the election would take place at all. Although only speculative, it is possible that this type of uncertainty dampens, rather than amplifies, the motivational force of identity. An interesting direction for future research would be to distinguish between different forms of political uncertainty and their effects on how identity and related factors shape political behavior.

To rule out alternative explanations, we compared trust in government to civic duty as an initial mediator. Although civic duty did mediate the relationship between national identity and voting, it did not form a serial chain with life satisfaction, suggesting that moral obligation alone may not confer the same psychological benefits as trust in government. In addition, all of our results held after controlling for various previously established sociodemographic and psychological predictors of voting.

Finally, we found that internal political self-efficacy moderated the serial indirect association between national identity and voting, with the effect being strongest among individuals low in political self-efficacy. This suggests that those with high political efficacy may rely more on their own sense of competence, while those with lower efficacy may depend more on external sources of motivation. This idea aligns with work by Caprara et al. (2009) and Niemi et al. (1991), who argue that trust in institutions can help compensate for low perceived political efficacy. It also fits with findings from Potoczek et al. (2023), who showed that individuals with low sociopolitical control (a construct related to political efficacy) are more influenced by ingroup norms and identity-based cues when deciding whether to vote. This also builds on Acar and Uluğ’s (2022) study, which hypothesized, but did not find, that political efficacy moderates an indirect link between trust and well-being. They speculated that in politically restricted contexts, political efficacy may be less influential. Still, our finding should be treated as tentative, given that the analysis was exploratory and not preregistered, and given the complexity and often limited reliability of interaction effects (Credé & Sotola, 2024).

### Limitations and Future Research

A key limitation of this study is the cross-sectional design and the measurement-of-mediation approach, meaning that our findings are entirely correlational. While the results seem quite robust given that they held both with and without theoretically relevant covariates, they do not tell us much about the causal order of the variables, and the results may be influenced by unmeasured confounding variables and measurement error (Pósch, 2021; Wilms et al., 2021). Although manipulating the constructs in our model might be challenging, future research could test how a national identity salience intervention impacts actual voter turnout (e.g. see Voelkel et al., 2024), as well as trust in government. While a brief national identity salience intervention is unlikely to influence broad, stable outcomes such as generalized trust in government or life satisfaction, it may affect more malleable state-like experiences, such as trust in a specific political process and momentary emotions. A more sustained intervention, such as a diary-based or longitudinal design that encourages ongoing reflection on one’s national identity from a secure and constructive perspective, may over time shape attitudes and intentions in ways that eventually influence real-world behavior, such as voter turnout.

Moreover, an important question is whether secure and defensive forms of national identity (Cichocka & Cislak, 2020) operate differently in shaping political behavior. Our study only included secure national identity, but it would be interesting to compare it to defensive national identification. Our re-analysis of the Kurdish data in the global collaborative COVID-19 project indicated that collective narcissism predicted prosocial behavior and attitudes positively, in the same direction as secure national identity. This seems to contradict previous research that has largely been conducted in WEIRD contexts. Unpacking such associations would be worthwhile.

Finally, the study was not preregistered, and the results should therefore be taken as preliminary. In future work, we aim to replicate and extend these findings and encourage further research to examine whether similar patterns emerge in other non-WEIRD contexts with comparable or distinct political and social structures.

## Conclusion

Our findings demonstrate how national identity can motivate civic engagement in a stateless region, even amid political uncertainty, economic instability, and widespread public disillusionment. A secure and constructive national identity was associated with voting intentions in regional elections despite multiple postponements, as well as actual voting in central government elections, even as turnout in the latter had fallen to a record low. Trust in government and life satisfaction emerged as key pathways linking identity to voting.

## Data Availability

Data, codebook, code (RMarkdown files and html-knitted RMarkdown files that contain both code and resulting output), and material (original language and English-translated versions) are available on the Open Science Framework (OSF) repository: https://osf.io/mvxwb/

## Notes

1. Due to a technical error during survey deployment by our research partner, the national identity items were presented on a three-point scale instead of the planned five-point scale. Nonetheless, the scale showed acceptable reliability (α = .79) and yielded results consistent with our hypotheses.

2. We initially considered including an item measuring whether participants leaned towards right-wing or left-wing politics but recognized that this distinction does not clearly apply to KRI, where political dynamics are shaped more by party allegiance and regional considerations than by a traditional left-right ideological divide. Thus, we decided not to include this. Moreover, this distinction is irrelevant to our study, as our focus is on voting, which in the Kurdish context is more influenced by party mobilization and political engagement than by ideological alignment

3. Blais and Galais (2016) measure civic duty using a four-item scale and construct an additive index by recoding responses into binary indicators and summing them, yielding a scale ranging from 0 to 3. We computed a continuous measure by taking the mean of the four items rather than summing binary-coded responses. This approach preserves variability in responses, allowing for finer distinctions in civic duty levels. Additionally, while Blais and Galais exclude respondents who selected “choice” from the first item, we retain them as the lowest level of civic duty. Excluding these respondents would result in significant data loss, which is unnecessary given that ambivalence or uncertainty about voting is meaningful in understanding civic duty.
